# Graft-derived cell free DNA: used for assessment of early graft status and its implications for long-term kidney function

**DOI:** 10.3389/fphys.2024.1440799

**Published:** 2024-11-15

**Authors:** Liang Wei, Yongheng Zhao, Shaoping Deng, Shaoping Wu, Hailian Wang, Xiangwei Luo, Hongji Yang

**Affiliations:** ^1^ Transplantation Health Management Center, Sichuan Taikang Hospital, Chengdu, Sichuan, China; ^2^ Department of Radiology, Sichuan Taikang Hospital, Chengdu, Sichuan, China; ^3^ Clinical Immunology Translational Medicine Key Laboratory of Sichuan Province, Sichuan Provincial People’s Hospital, School of Medicine, University of Electronic Science and Technology of China, Chengdu, China; ^4^ Transplantation Center, Sichuan Provincial People’s Hospital, School of Medicine, University of Electronic Science and Technology of China, Chengdu, China

**Keywords:** graft-derived cell-free DNA, kidney transplantation, estimated glomerular filtration rate, EGFR, kidney graft function

## Abstract

**Background:**

The long-term graft survival is closely related to its early status, yet the indices for assessing the early graft status are complex and lack quantitative values. The aim of this study is to investigate the potential of GcfDNA as a comprehensive, non-invasive, convenient, and quantifiable indicator for evaluating early graft status.

**Methods:**

In this study, 138 recipients who underwent primary kidney transplantation were enrolled. Peripheral blood samples, each 10 mL, were collected on days 1 and 7 post-transplantation. The quantification of both the graft cell-free DNA (GcfDNA) fraction (%) and GcfDNA concentration (copies per milliliter, cp/mL) was performed using droplet digital PCR (ddPCR).

**Results:**

For most recipients, both the GcfDNA fraction and concentration had a rapid decline at 7 days post-transplantation, reaching median values of approximately 0.7% and 53.5 cp/mL, respectively. No significant associations were found between GcfDNA values and other clinical parameters. On the seventh postoperative day, we observed a significant elevation in GcfDNA concentration among recipients with eGFR values < 60 mL/min/1.73 m^2^. Additionally, notable increases were identified in both GcfDNA fraction and concentration variations within this specific subgroup. The findings of our study indicate a negative correlation between the concentration and fractional changes of GcfDNA on postoperative days 1 and 7, as well as the GcfDNA concentration on postoperative day 7, with eGFR within the 1–2 years post-transplantation period. The ROC curve of GcfDNA_Copies_Variation. day1-day 7 showed the highest AUC value AUC = 0.8006, with high sensitivity (90.14%) and specificity (77.61%), and PPV and NPV were 81.01% and 88.14%, respectively. Using four classical algorithm models, we found that the xgboost regression model achieved the best predictive performance (area under the curve (AUC) values = 0.862) for eGFR within 1–2 years post-transplantation, with high sensitivity (85.7%) and specificity (85%).

**Conclusion:**

The changes of GcfDNA levels in the early stage are closely related to kidney function within 1–2 years post-transplantation. As a comprehensive indicator of graft function, GcfDNA has great potential for clinical application.

## Highlights

> At 7 days post-transplantation, a significant reduction was noted in both the GcfDNA fraction and concentration among recipients exhibiting eGFR values below 60 mL/min/1.73 m^2^.
> The ROC curve analysis of GcfDNA copies variation from day 1 to day 7 demonstrated the highest AUC value of 0.8006.
> The xgboost regression model achieved the best prediction effect (AUC = 0.862) for eGFR within 1–2 years post-transplantation.

## Introduction

Kidney transplantation represents the optimal treatment modality for patients with end-stage kidney disease (ESKD). However, due to organ shortages, donor kidneys of suboptimal quality are being transplanted. Although advancements in surgical techniques and the introduction of novel immunosuppressive regimens have notably enhanced graft survival rate (Wang et al., 2016). Currently, the one-year kidney allograft survival rate exceeds 95%; however, the long-term viability of transplanted kidneys remains suboptimal, with graft failure rates of approximately 10%–20% at 5 years, and only 51%–69% at 10 years (Hart et al., 2021). Long-term kidney graft survival is influenced by numerous short-term factors such as donor and recipient ages, HLA mismatch, cold ischemia time (CIT), surgical expertise, comorbidities, among others (Wu et al., 2017; Dziewanowski et al., 2018; Senev et al., 2020). For instance, the employment of the estimated glomerular filtration rate (eGFR) is recommended for identifying renal allograft dysfunction according to the KDIGO (Kidney Disease: Improving Global Outcomes) guidelines (Kasiske et al., 2010). Research has indicated that donor age exceeding 70 years, and recipient ages either below 29 or above 57, are correlated with a one-year reduction in eGFR (Hong et al., 2022). An HLA mismatch constitutes a risk factor for reduced eGFR within the first post-transplantation year (Foroutan et al., 2019). Prolonged CIT, particularly following circulatory death (DCD) or brain death (DBCD), correlates with a statistically significant decrease in eGFR within the first post-transplantation year, with an average hourly reduction of 0.7 mL/min/1.73 m^2^ (Luo et al., 2021). This implies that multiple factors contribute to the long-term functionality of the graft, making a comprehensive assessment of graft health challenging. Consequently, there is an urgent need to identify a comprehensive and universal clinical biomarker capable of evaluating long-term kidney graft function during the early post-transplantation period.

In recent years, numerous studies have substantiated the potential of graft-derived cell-free DNA (GcfDNA) as a promising non-invasive biomarker for monitoring allograft injury, acute and chronic rejection following solid organ transplantation, including liver, lung, heart, and kidney (Khush, 2021; Garg et al., 2021; Schütz et al., 2017; Jang et al., 2021). It primarily consists of fragmented DNA, with lengths ranging from 120 to 160 base pairs, which is released from cells experiencing damage within the allograft due to injury or rejection (Thongprayoon et al., 2020; Whitlam et al., 2019). Although GcfDNA concentrations are generally low in various body fluids like plasma, serum, and urine, spanning from a few hundred to several thousand genomic copies per milliliter (Whitlam et al., 2019; Oellerich et al., 2019), they can still be detected using droplet digital PCR (ddPCR) or targeted next-generation sequencing (NGS) (Knight et al., 2019). Within this field of study, most research has focused on exploring the potential application of GcfDNA as a non-invasive biomarker for graft rejection (Yang et al., 2024). The outcomes are encouraging, in several prospective studies, nearly 70% of recipients exhibited elevated levels of GcfDNA fraction, detectable up to a month before the clinical diagnosis of rejection (Knight et al., 2019; García Moreira et al., 2009). Additionally, Hinojosa et al. indicated that GcfDNA has the potential for real-time monitoring of the response to acute rejection treatment (Hinojosa et al., 2019). A KidneyCare platform, developed by Gray et al., suggested that GcfDNA could aid in optimizing the immunosuppressive regimen (Gray et al., 2020). It was also found that recipients with delayed graft function (DGF) typically exhibit higher levels of GcfDNA fraction shortly after transplantation (Shen et al., 2019; Di et al., 2021). Thus, GcfDNA is considered a useful biomarker for early, non-invasive, real-time monitoring of allograft injury with high specificity, which could avoid unnecessary biopsies and allow early therapeutic intervention.

The fundamental principle underpinning the early diagnosis of transplant injury via GcfDNA involves detecting cell-free DNA (cfDNA) originating from the transplanted cells. Regardless of the cause of transplant injury, a notable increase in GcfDNA can be observed in the recipient’s bloodstream. Studies have indicated an association between elevated GcfDNA levels and the load of HLA class II eplet mismatches (González-López et al., 2023). This investigation pioneers the exploration of the linkage between HLA class II eplet mismatches and heightened GcfDNA concentrationsFurthermore, Els M. Gielis and colleagues identified a relationship among donor and recipient ages, HLA mismatches, CTI, and median baseline GcfDNA levels (Gielis et al., 2018). It demonstrates a connection between GcfDNA levels and various elements affecting kidney graft functionality over the long term. As reported by some studies, the higher the damage verified by GcfDNA, the higher the likelihood that kidney function worsens over time, regardless of the cause (Bu et al., 2022; Stites et al., 2020). Hence, employing GcfDNA as an all-encompassing marker for consistently evaluating kidney function over the long term appears promising for clinical use. According to our knowledge, only a few studies have explored the link between GcfDNA fraction (%), concentration (cp/mL), its rate of change, and kidney graft function in the long term.In a previous study, we utilized a classical random forest regression model to forecast eGFR 90 days after kidney transplantation based on GcfDNA fraction and various clinical parameters, achieving positive predictive results (Di et al., 2021). We then evaluated the connection between early post-transplantation GcfDNA anomalies and renal graft function in the early stage. Our research suggests that measuring GcfDNA shortly after transplantation may offer a new method to assess the short-term threat of compromised kidney allograft function or DGF (Yang et al., 2022). This investigation links early GcfDNA levels with kidney function markers during the 1–2 years following transplantation, investigating its capacity as an comprehensive, non-invasive, convenient, and quantifiable indicator for evaluating early graft status.

## Materials and methods

### Patients and samples

Ethical approval was obtained from the Sichuan Provincial People’s Hospital under reference number 2023281, and all participants provided written informed consent after full disclosure of the study details. Enrolled in the study from April 2018 to September 2020 were adult recipients of deceased donor kidneys who had undergone primary kidney transplantation. The exclusion criteria included patients who underwent multi-organ transplantation, those who died, and those lost to follow-up. Out of 174 assessed recipients, 138 were included in the study. Data collection continued until September 2021, which led to varying follow-up durations for each participant.

### Blood collection and DNA extraction

At day 1 and day 7 post-transplantation, 10 mL peripheral blood samples were collected from each recipient. DNA was extracted using the method detailed in the previous study (Di et al., 2021).

### Quantification of GcfDNA

Fractional abundance (%) and genomic copies per mL plasma (cp/mL) of GcfDNA were quantified using the YiLeShu-Graft Sentinel® service from SKM Biotechnology Co., Ltd. (Di et al., 2021). The assay was conducted utilizing the Bio-Rad QX200 Droplet Digital System (Bio-Rad Laboratories, US), and data processing was performed with QuantaSoft™ version 1.7.4 software (Bio-Rad Laboratories, US). Assay workflow specifications were detailed in our previous publication (Di et al., 2021).

### Statistical analysis

The statistical analyses were conducted using R version 4.0.3 software and GraphPad Prism 9.1 software. The Mann-Whitney U test or Chi-square test was used to evaluate differences that are significant in clinical variables among different groups. The association and correlation between variables, notably eGFR from 1 to 2 years post-transplantation, and the values of GcfDNA fraction (%) and GcfDNA concentration (cp/mL) on postoperative days 1 and 7, along with their changes, were analyzed using Pearson’s correlation test. Receiver operating characteristic (ROC) curves were used for calculating the area under the curve (AUC) with a 95% confidence interval (CI), and the classification effects on kidney function changes in recipients were also assessed. A two-tailed *P*-value less than 0.05 was deemed statistically significant.

## Results

**FIGURE 1:**
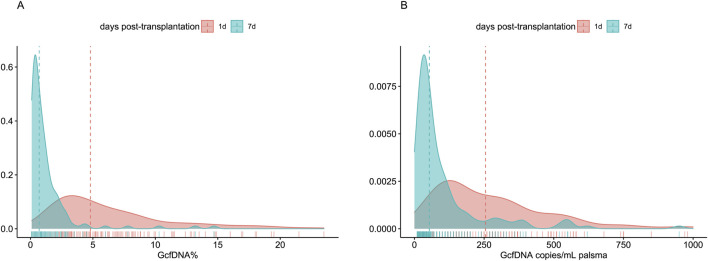
The distribution profile of GcfDNA fraction and concentration on days 1 and 7 post-transplantation. **(A)** The distribution profile of GcfDNA fraction (%). **(B)** The distribution profile of GcfDNA concentration (copies/mL). The *y*-axis on each graph indicated the probability density.

**FIGURE 2:**
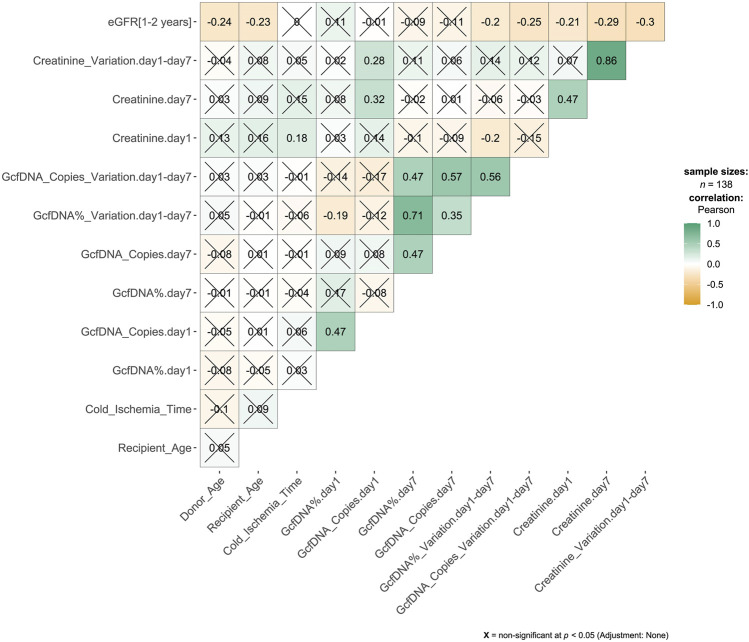
Correlation matrix results from pairwise Pearson correlation test. The association between variables was analyzed by Pearson’s correlation test. The number in the cell of the matrix was the Pearson correlation coefficient, the orange to green coloring in the cells of the matrix indicated an increase of the number.

A total of 276 plasma samples were collected from 138 recipients. The median GcfDNA fraction was 4.8%, and the median GcfDNA concentration was 255 cp/mL on the first day post-transplantation (Figure 1). On the seventh day post-transplantation, both the GcfDNA fraction and GcfDNA concentration exhibited a rapid decline, reaching median values of approximately 0.7% and 53.5 cp/mL, respectively (Figure 1). The clinical characteristics, encompassing donor age, recipient age, recipient sex, HLA mismatches, cold ischemia time, as well as the values of GcfDNA fraction, GcfDNA concentration, and creatinine on postoperative days 1 and 7, the variations of relevant clinical variables (the ratio of creatinine, GcfDNA fraction, and concentration at 7 days post-transplantation to those of the first day), and the values of eGFR within 1–2 years post-transplantation, were summarized in Table 1. No significant associations were identified among these clinical parameters (Figure 2).

**TABLE 1:** Recipient and donor characteristics.

	Overall (N = 138)
Recipient_Sex	
Female	38 (27.5%)
Male	100 (72.5%)
Donor_Age (years)	
Mean (SD)	46.6 (13.6)
Recipient_Age (years)	
Mean (SD)	39.3 (10.1)
HLA_Mismatches	
2	5 (3.6%)
3	22 (15.9%)
4	50 (36.2%)
5	47 (34.1%)
6	14 (10.1%)
Cold_Ischemia_Time (mins)	
Mean (SD)	485 (190)
GcfDNA%.day1	
Mean (SD)	6.36 (4.74)
GcfDNA_Copies.day1 (copies/mL plasma)	
Mean (SD)	429 (681)
GcfDNA%.day7	
Mean (SD)	1.30 (2.06)
GcfDNA_Copies.day7 (copies/mL plasma)	
Mean (SD)	132 (270)
GcfDNA%_Variation.day1-day7	
Mean (SD)	0.31 (0.51)
GcfDNA_Copies_Variation.day1-day7	
Mean (SD)	0.65 (1.32)
Creatinine.day1 (μmol/L)	
Mean (SD)	880 (359)
Creatinine.day7 (μmol/L)	
Mean (SD)	372 (374)
Creatinine_Variation.day1-day7	
Mean (SD)	0.41 (0.33)
eGFR[1–2 years] (mL/(min·1.73 m^2))	
Mean (SD)	63.3 (26.4)
eGFR[1–2 years] (mL/(min·1.73 m^2))	
Median (SD)	60.6 (22.3)
eGFR tests/pt/1–2 years (NO.)	
Mean (SD)	25 (6)
eGFR tests post-transplant (days)	
Median (SD)	155 (29.3)
Mean (SD)	216.4 (35.6)
Follow-up interval [<3 months] (days)	
Median (SD)	10 (5)
Follow-up interval [3–6 months] (days)	
Median (SD)	19 (9)
Follow-up interval [7–12 months] (days)	
Median (SD)	29 (11)
Follow-up interval [>12 months] (days)	
Median (SD)	55 (19)

**FIGURE 3:**
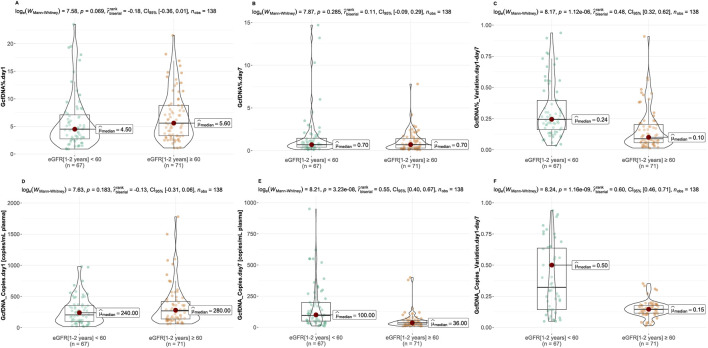
Comparison of GcfDNA variables by cohort. **(A, B)** Comparison of GcfDNA fraction on postoperative day 1 and 7 between the healthy and impaired kidney function groups. **(C)** Comparison of the variations of GcfDNA fraction between two groups. **(D, E)** Comparison of GcfDNA concentration on postoperative day 1 and 7 between two groups. **(F)** Comparison of the variations of GcfDNA concentration between two groups. *P* values were calculated using the Mann-Whitney U test. A *P*-value < 0.05 was considered to be statistically significant.

**FIGURE 4:**
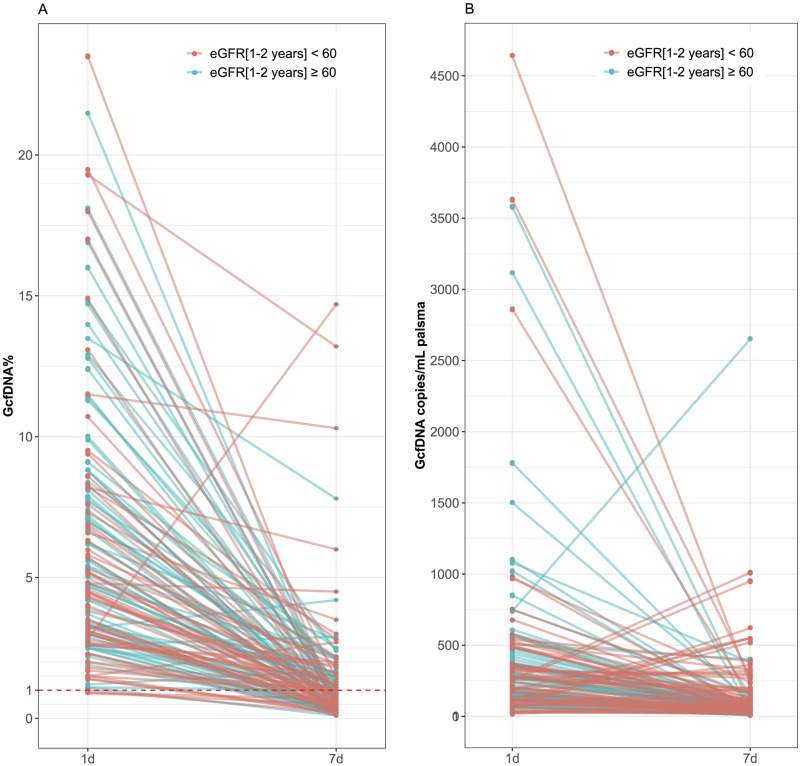
Comparison of GcfDNA fraction and concentration on postoperative day 1 and day 7 in all recipients by cohort. **(A)** Comparison of GcfDNA fraction on postoperative day 1 and day 7. **(B)** Comparison of GcfDNA concentration on postoperative days 1 and 7. Note that decreases in GcfDNA fraction and concentration were observed in most recipients with healthy kidney function.

Recipients were classified into healthy and impaired kidney function groups based on their eGFR values, where a value less than 60 mL/min/1.73 m^2^ is considered as a clinically relevant decrease in kidney function, according to the KDIGO 2012 Clinical Practice Guideline. As depicted in Table 2, our findings show statistically significant differences between groups in several traditional clinical variables, such as recipient age, creatinine levels on postoperative day 7, and its variation (*p* < 0.05). While no significant differences were observed in GcfDNA fraction between groups (Figure 3), the GcfDNA concentration on postoperative day 7, and the variations in both GcfDNA fraction and concentration were found to be higher in the impaired kidney function group (Figure 3). Through comparing the GcfDNA fraction and concentration on postoperative day 1 and day 7 in all recipients, we noted that most recipients exhibited decreases in these two variables with eGFR values ≥ 60 mL/min/1.73 m2 (Figure 4).

**TABLE 2:** Recipient and donor characteristics between different groups.

	eGFR[1-2 years] < 60	eGFR[1-2 years] ≥ 60	*P*-value
	(N = 67)	(N = 71)	
Recipient_Sex			
Female	23 (34.3%)	15 (21.1%)	0.12
Male	44 (65.7%)	56 (78.9%)	
Donor_Age (years)			
Mean (SD)	49.0 (13.2)	44.3 (13.8)	0.087
Recipient_Age (years)			
Mean (SD)	41.2 (9.32)	37.5 (10.6)	<0.05
HLA_Mismatches			
2	4 (6.0%)	1 (1.4%)	<0.05
3	16 (23.9%)	6 (8.5%)	
4	20 (29.9%)	30 (42.3%)	
5	22 (32.8%)	25 (35.2%)	
6	5 (7.5%)	9 (12.7%)	
Cold_Ischemia_Time (mins)			
Mean (SD)	494 (225)	477 (151)	0.55
GcfDNA%.day1			
Mean (SD)	5.86 (4.92)	6.84 (4.54)	0.069
GcfDNA_Copies.day1 (copies/mL plasma)			
Mean (SD)	421 (765)	436 (598)	0.18
GcfDNA%.day7			
Mean (SD)	1.59 (2.68)	1.03 (1.17)	0.29
GcfDNA_Copies.day7 (copies/mL plasma)			
Mean (SD)	178 (205)	89.7 (315)	<0.05
GcfDNA%_Variation.day1-day7			
Mean (SD)	0.37 (0.68)	0.21 (0.28)	<0.05
GcfDNA_Copies_Variation.day1-day7			
Mean (SD)	1.11 (1.73)	0.21 (0.44)	<0.05
Creatinine.day1 (μmol/L)			
Mean (SD)	905 (341)	856 (377)	0.32
Creatinine.day7 (μmol/L)			
Mean (SD)	469 (375)	280 (351)	<0.05
Creatinine_Variation.day1-day7			
Mean (SD)	0.51 (0.33)	0.32 (0.31)	<0.05

**FIGURE 5:**
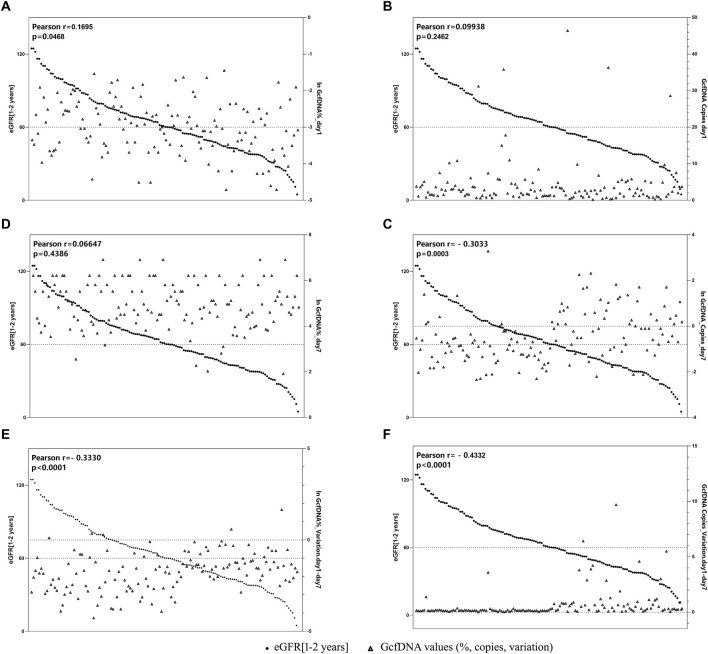
The correlation between GcfDNA levels in early period with eGFR at 1–2 years. **(A, B)** Correlation between GcfDNA levels on postoperative day 1 with eGFR. **(C, D)** Correlation between GcfDNA levels on postoperative day 7 with eGFR. **(E, F)** Correlation between the change rate of GcfDNA levels from day 1 to day 7 after postoperative with eGFR.

Figure 5 illustrates the correlation between eGFR within 1–2 years post-transplantation and the values of GcfDNA fraction (%) and GcfDNA concentration (cp/mL) on postoperative days 1 and 7, along with these variations. Our findings indicate a negative correlation between the concentration and fractional (ln) changes of GcfDNA on postoperative days 1 and 7, as well as the GcfDNA concentration (ln) on postoperative day 7, with eGFR within 1–2 years post-transplantation (Figure 5).

**FIGURE 6:**
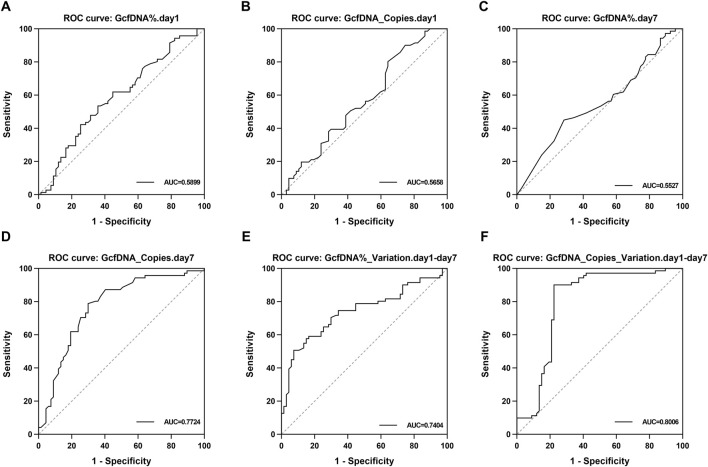
The evaluate performance of GcfDNA at different times and values. **(A–F)** The ROC analysis for GcfDNA fraction (%), concentration (cp/mL), and rate of change on postoperative days 1 and 7. The AUC with 95% CI was calculated. GcfDNA_Copies_Variation.day1-day7, GcfDNA_Copies.day 7 and GcfDNA%_Variation.day1-day 7 have the better ROC curve and AUC value.

Upon applying receiver operating characteristic (ROC) curve analysis, we observed results with high sensitivity and specificity (Figure 6; Table 3). The ROC curve for GcfDNA_Copies_Variation.day1-day7 revealed the highest AUC value (0.8006, 95% confidence interval [CI] = 0.7181–0.8831). An optimal cut-off value of 0.2050 resulted in a sensitivity of 90.14% and a specificity of 77.61%. The positive predictive value (PPV) and negative predictive value (NPV) were 81.01% and 88.14%, respectively. Furthermore, the ROC curve of GcfDNA_Copies.day7 revealed an AUC value at 0.7724 (95% confidence interval [CI] = 0.6919–0.8530), with a sensitivity measured at 78.87%, a specificity at 70.15%, and the PPV and NPV at 73.68% and 75.81%, respectively. The ROC curve of GcfDNA%_Variation.day1-day7 revealed an AUC value at 0.7404 (95% confidence interval [CI] = 0.6564–0.8244), with a sensitivity measured at 50.70%, the specificity at 92.54%, and the PPV and NPV at 87.80% and 63.92%, respectively.

**TABLE 3:** The classification effect of each classifier on recipients of renal function changes.

Feature	Cut off	AUC	Sensitivity (%)	Specificity (%)	PPV	NPV
GcfDNA%.day1	0.0535	0.5899	53.52%	64.18%	61.29%	56.58%
GcfDNA_Copies.day1	1.2500	0.5658	80.28%	35.82%	57.00%	63.16%
GcfDNA%.day7	0.0045	0.5527	45.07%	71.64%	62.75%	55.17%
GcfDNA_Copies.day7	0.5950	0.7724	78.87%	70.15%	73.68%	75.81%
GcfDNA%_Variation.day1-day7	0.1007	0.7404	50.70%	92.54%	87.80%	63.92%
GcfDNA_Copies_Variation.day1-day7	0.2050	0.8006	90.14%	77.61%	81.01%	88.14%

**FIGURE 7:**
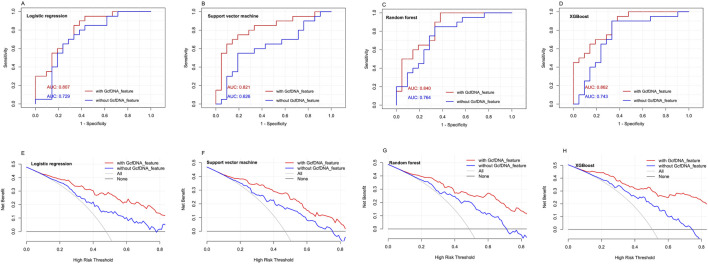
The predictive performance of different models with or without GcfDNA variables. **(A-D)** ROC analysis for multivariate logistic regression model, random forest regression model, xgboost regression model and rpart regression model with or without GcfDNA variables was used to predict the risks of impaired kidney function within 1 to 2 years post-transplantation. The AUC with 95% CI was calculated. **(E-H)** DCA were established to evaluate the predictive value of the four classical models with or without GcfDNA variables.

Using the four classical models, including multivariate logistic regression model, random forest regression model, xgboost regression model and rpart regression model, our results revealed that the use of xgboost regression model obtained the highest area under the curve (AUC) values (0.862, 95% confidence interval [CI] = 0.758–0.994) when predicting eGFR within 1–2 years post-transplantation. An optimal cut-off value was indicated at 0.609, with a sensitivity of 85.7% and a specificity of 85% (Figure 7). Decision curve analysis (DCA) indicated that xgboost regression model provided a larger net benefit compared with other models (Figure 7). And it also suggested that the four models had better predicted values with GcfDNA parameter compared with those without it (Figure 7).

## Discussion

In this study, we observed a significant increase in GcfDNA levels during the first 1–7 days post-transplantation, which was associated with the decline in eGFR within 1–2 years after transplantation. These findings emphasize the importance of early GcfDNA as a monitoring tool for renal function and highlight the necessity of paying attention to biomarker changes in the early post-transplantation. These results are crucial for enhancing understanding of the clinical value of GcfDNA as a novel biomarker. In the subsequent discussion, we will delve into a detailed analysis of these observations and contextualize them within the current literature, aiming to further explore the potential of GcfDNA as a comprehensive indicator.

Many previous studies have regarded GcfDNA as a novel and independent diagnostic or prognostic biomarker (Gray et al., 2020; Shen et al., 2019; Wijtvliet et al., 2020). In our opinion, it could be used for real-time monitoring or evaluating various aspects of kidney graft health, not only by focusing on the values of GcfDNA at a single time point, but also by considering the patterns of change over a period. In the present study, we firstly used the variations of GcfDNA fraction and concentration in the 7 days post-transplantation as the clinical parameter. We observed that, for most recipients, there was a rapid decline in GcfDNA fraction within 7 days, consistent with many previous studies (Oellerich et al., 2019; Shen et al., 2019; Di et al., 2021), and we also noted a similar decreasing trend in GcfDNA concentration as well as in GcfDNA fraction. The abnormally high values at early clinical time points after transplantation are thought to be associated with ischemia/reperfusion injury (IRI) (Gielis et al., 2018). Moreover, no significant associations were found between GcfDNA and other clinical variables, indicating that GcfDNA was a relatively independent biomarker. Some scholars have also suggested that early GcfDNA levels can serve as a predictive indicator for the long-term function of a transplanted kidney (Cucchiari et al., 2023). Evaluating renal function in transplant recipients is crucial for assessing graft health and overall patient outcomes. eGFR provides a valuable estimate of the kidney’s filtration efficiency and is widely recognized as a reliable indicator in renal transplantation research. Its application allows for the timely detection of changes in renal function, aiding in the identification of potential issues such as graft rejection or dysfunction. In our study, we utilized the eGFR as a key metric to gauge renal function within the first 1–2 years post-transplantation. Our findings suggest that, although there were no significant differences in GcfDNA fraction between healthy recipients and those with impaired kidney function (regardless of the cause), the variations in both GcfDNA fraction and concentration were significantly higher in recipients with eGFR values lower than 60 mL/min/1.73 m^2^. Therefore, continuous monitoring of the changes in GcfDNA values may play an important role in clinical practice. It should be noted that we did not analyze the results of biopsy punctures in these recipients within 1–2 years. This decision was made due to the inherent risks associated with biopsy procedures, and it is uncommon for recipients to voluntarily opt for biopsy puncture testing.

The study by Bu et al. demonstrated an association between increased GcfDNA levels and a significant decrease in eGFR 3 years post-transplantation, indicating that higher GcfDNA levels may heighten the risk of eGFR decline (Bu et al., 2022). The study conducted by Cucchiari D et al. revealed an association between 24-h GcfDNA and 6-month eGFR, with a correlation coefficient of −0.311 and a *P*-value of 0.023 (Cucchiari et al., 2023). Based on these findings and the broader context of existing research, we expanded our analysis to investigate the relationship between GcfDNA levels within the first 1–7 days post-transplantation and eGFR within the following 1–2 years. Our investigation found a correlation: patients with higher GcfDNA levels during the initial 1–7 days post-transplant experienced a greater reduction in eGFR within 1–2 years. This finding further substantiates the link between GcfDNA levels in the early stage and long-term renal function. Recently, research has employed short-term GcfDNA (24-h) as a predictor of long-term (6-month) kidney function. It was reported that patients with 24-h GcfDNA levels in the lowest quartile (<1.78%) exhibited superior kidney function at the six-month mark compared to those in other quartiles (Gielis et al., 2018). This suggests that identifying injury before traditional functional changes occur may have significant implications for the long-term survival of the graft. Building upon these observations, we further explored the corresponding receiver operating characteristic (ROC) curve. It is noteworthy that the correlation between the natural logarithm of changes in GcfDNA concentration (cp/mL) within 1–2 years post-transplantation and eGFR is relatively high in our study, accompanied by correspondingly elevated AUC values. Oellerich et al. compared accuracy of GcfDNA fraction (%) and GcfDNA concentration (cp/mL) in detecting graft injury. The ROC curves for GcfDNA (cp/mL) and GcfDNA (%) showed the superior performance (*p* = 0.02) of the absolute amount (cp/mL) of GcfDNA rather than the GcfDNA fraction (%) (Oellerich et al., 2019), indicating that using absolute quantification in copies per mL plasma may avoid biasing influence (e.g., apoptotic leukocytes, infections, exercise, non-graft-associated vascular compromise, medications) (Sun et al., 2015). Some scholars have pointed out that both the percentage and absolute values of GcfDNA have their own strengths and limitations (De Vlaminck, 2020). Integrating these two values can provide a more comprehensive presentation of information (Whitlam et al., 2019; Oellerich et al., 2019; De Vlaminck, 2020).

Early diagnosis could greatly improve the survival of kidney recipients. However, the diagnostic methods currently employed in clinical practice have limitations due to being either invasive or insensitive. As a promising non-invasive biomarker, GcfDNA has been extensively studied for its role in the clinical diagnosis of rejection, particularly antibody-mediated rejection (ABMR). In a systematic review, Wijtvliet et al. systematically assessed 14 eligible studies and performed a meta-analysis. The results revealed that kidney transplant recipients with ABMR had significantly higher GcfDNA fractions compared to those without rejection or in stable condition (Wijtvliet et al., 2020). Despite presenting higher GcfDNA levels in patients with ABMR, allograft injuries resulting from other causes such as infection, surgical complications, etc., can also lead to changes in GcfDNA levels (Gielis et al., 2018). Gielis et al. further supported this viewpoint, demonstrating that an increase in GcfDNA percentage above the threshold of 0.88% not only correlated with acute rejection (*P* = 0.017) but also significantly associated with the occurrence of acute tubular necrosis (*p* = 0.011) and acute pyelonephritis (*P* = 0.032) (Gielis et al., 2020). Therefore, GcfDNA serves as a sensitive biomarker not only for rejection but also for various types of allograft injury. We acknowledge that factors influencing long-term graft function are diverse and complex. It is insufficient to assess long-term graft function solely through a single indicator. Nevertheless, existing evidence suggests that GcfDNA holds promise as an early comprehensive indicator, offering early warning for the long-term changes in allograft function. In the present study, through using four classical algorithm models, we found that the use of xgboost regression model could achieve good predictive performance for eGFR within 1–2 years post-transplantation, with high sensitivity and specificity. Thus, GcfDNA is a valuable marker that can assist clinicians in the post-transplant management of patients, including individualized immunomodulation and graft function monitoring. The integration of GcfDNA as a biomarker into a more comprehensive approach to post-transplant care, combined with other clinical and laboratory parameters, has the potential to optimize patient outcomes.

Despite these advances, there are still some limitations and challenges. We only used surrogate markers of renal injury (eGFR) as the evaluation criterion for graft function, lacking relevant data for biopsy and puncture. In addition, although the number of recipients recruited from a single-hospital is a limitation of conclusion in this study, our results suggested a novel strategy and possible way to evaluate various complications and adverse events after transplantation. As the multi-center clinical studies with larger sample size, long-term and continuous monitoring, and more comprehensive clinical data, are conducted in the future, it holds promise that real-time and personalized graft health monitoring after kidney transplantation could be realized in clinical application, and it will improve the long-term survival of kidney allograft.

## Conclusion

In conclusion, our study suggests that the changes of GcfDNA level in the early stage are closely related to kidney function within 1–2 years post-transplantation. GcfDNA has great potential to serve as a comprehensive indicator of graft function.

## Data Availability

The original contributions presented in the study are included in the article/supplementary material, further inquiries can be directed to the corresponding author.
